# Nitrate Is Crucial for the Proliferation of Gut *Escherichia coli* Caused by H9N2 AIV Infection and Effective Regulation by Chinese Herbal Medicine Ageratum-Liquid

**DOI:** 10.3389/fmicb.2020.555739

**Published:** 2020-10-30

**Authors:** Xinheng Zhang, Qiqi Zhao, Che Wu, Zi Xie, Xiaotong Ci, Hongxin Li, Wencheng Lin, Huanmin Zhang, Qingmei Xie

**Affiliations:** ^1^College of Animal Science, South China Agricultural University, Guangzhou, China; ^2^Lingnan Guangdong Laboratory of Modern Agriculture, Guangzhou, China; ^3^Guangdong Engineering Research Center for Vector Vaccine of Animal Virus, Guangzhou, China; ^4^South China Collaborative Innovation Center for Poultry Disease Control and Product Safety, Guangzhou, China; ^5^USDA, Agriculture Research Service, Avian Disease and Oncology Laboratory, East Lansing, MI, United States

**Keywords:** H9N2 AIV, gut microbiota, *E. coli*, secondary infection, metabolite, nitrate, ageratum-liquid

## Abstract

H9N2 avian influenza virus (AIV) infection in chickens is often accompanied by secondary bacterial infection, but the mechanism is unclear. The aim of the present study was to reveal that mechanism and explore non-antibiotic treatment. 16s rRNA sequencing and metabonomics were performed in the intestinal contents of chickens infected with H9N2 AIV or H9N2 AIV and fed with ageratum-liquid (AL) to reveal the metabolite that promote intestinal *Escherichia coli (E. coli)* proliferation caused by H9N2 AIV, as well as to determine the regulatory effect of AL. It was found that H9N2 AIV infection led *E. coli* to become the dominant gut microbe and promoted *E. coli* translocation from the intestinal tract to the visceral tissue through the damaged intestinal barrier. H9N2 AIV infection induces inflammation in the intestinal mucosa and promotes the secretion and release of nitrate from the host intestinal epithelium. In addition, nitrate promoted *E. coli* proliferation in the inflamed intestinal tract following H9N2 AIV infection. Furthermore, Chinese herbal medicine AL can restore intestinal homeostasis, inhibit the production of nitrate in the intestinal epithelium and effectively prevent the proliferation and translocation of *E. coli* in the intestines. This is the first report on the mechanism of *E. coli* secondary infection induced by H9N2 AIV, where herbal medicine AL was shown to have a good preventive effect on the secondary infection.

## Introduction

Avian influenza virus (AIV) belongs to the influenza virus A genus of the Orthomyxoviridae family (Marc, 2016). The genetic material of AIV is composed of single-stranded negative-stranded RNA, which is divided into 8 independent fragments (Resa-Infante et al., 2011). AIV is divided into the H1-16 and N1-9 subtypes, according to the antigenic types of hemagglutinin (HA) and neuraminidase on the envelope (Lee et al., 2016). Due to the different pathogenicity of the different subtypes, AIV can have high and low pathogenicity. Highly pathogenic AIV mainly includes the H5 and H7 subtypes and low pathogenic AIV the H9N2 subtype (Gharaibeh, 2008; Pantin-Jackwood and Swayne, 2009).

H9N2 AIV is widespread in all parts of the world and was first isolated from turkeys in 1966 (Peacock et al., 2019). The biggest harm of H9N2 AIV infection in poultry is the induction of immunosuppression and destruction of their immune system. H9N2 AIV can be isolated from host tissues, including the trachea, lung, brain, spleen, pancreas, cloacal cavity and intestinal tract, and causes inflammation and enteric problems to the hosts (Li et al., 2018). H9N2 AIV is prone to secondary infection of bacteria and other pathogens, the most common of which is *Escherichia coli (E. coli)* infection, which is associated with high mortality in poultry (Barbour et al., 2009; Samy and Naguib, 2018; Nguyen et al., 2019). The H9N2 AIV subtype poses a significant public health threat, as it can replicate in permissive mammalian tissues without prior adaptation (Samir et al., 2019). Furthermore, several influenza viruses that infect humans all originate from variants of H9N2 AIV (Lam et al., 2013; Chen et al., 2014); hence, the potential harmful effects of H9N2 AIV on aquaculture and human health and safety cannot be ignored.

A large number of studies have shown that viral infections can destroy the balance of intestinal microorganisms and cause inflammation (Kotler et al., 1993; Groves et al., 2018; Kritas et al., 2020). The influenza virus can not only change the respiratory tract flora, but also affect the composition of intestinal microorganisms located far away from the respiratory tract; the reason for that is not clear (Qin et al., 2015). So far, only a few articles have reported that H9N2 AIV infection can affect poultry intestinal microorganisms (Yitbarek et al., 2018a,c). Previously, H9N2 AIV was reported to affect intestinal microbiota, barrier structure injury and inflammatory intestinal disease in the chicken ileum, but the mechanism of H9N2 AIV-induced *E. coli* proliferation remains unclear (Li et al., 2018). It was recently reported that the intestinal epithelium of the host produces certain active substances under the condition of inflammation to provide growth factors for conditional pathogenic bacteria in the intestinal tract (Haberman et al., 2014). The metabolic substances of the microbiota can affect the immune homeostasis, but also the sensitivity of the host to several immune-mediated diseases, and the regulatory substances of the host epithelium can provide a colonization environment for the microbiota (Rooks and Garrett, 2016). Gut microbiota uses their substrates to generate energy for cellular processes and for growth, the microbiota produces several metabolites that influence human health and metabolism (Ramakrishna, 2013). We therefore speculated that intestinal metabolites also play a key role in inducing the proliferation of *E. coli* in H9N2 AIV-infected chickens. Most importantly, to date, there has been no report on the relationship between intestinal metabolism and intestinal microbiota disorder caused by H9N2 AIV.

H9N2 infection can induce inflammation in chickens. Antibiotics are often used in the clinic to treat inflammation; however, although antibiotics can kill bacteria, they can also cause damage to the beneficial flora of the intestine, aggravating intestinal injury due to its broad spectrum (Li et al., 2018). A large number of studies have shown that Chinese herbal medicine can have a favorable effect on the regulation of the intestinal tract; an example of such medicine is ageratum-liquid (AL), which has been shown to exert a significant curative effect against gastrointestinal influenza (Lu et al., 2011; Liu et al., 2014). AL has been shown to regulate CD4^+^ and CD8^+^ cells in Peyer’s patch and suppress tumor necrosis factor (TNF)-α levels in enteric homogenates to improve the diarrhea caused by *Salmonella typhimurium* in mice (He et al., 2006; Ko et al., 2011; Chen et al., 2016). Our previous study showed that AL can effectively prevent bacterial translocation following H9N2 infection in mice (Lu et al., 2019). In our previous study, it was also demonstrated that H9N2 AIV infection could affect intestinal microbiota, barrier structure injury, and inflammatory intestinal disease in the chicken ileum (Li et al., 2018). We therefore aimed at exploring whether AL has a good therapeutic effect on intestinal flora disorder and bacterial infection induced by H9N2 AIV in chickens.

The purpose of this study was to explore the relationship between intestinal flora disorder and intestinal metabolism, in order to reveal the cause of secondary *E. coli* infection induced by H9N2 AIV infection in chickens. In addition, through the identification of the efficacy of AL in the treatment of intestinal flora disorder, inflammation and intestinal bacterial translocation induced by H9N2 AIV infection, the new data for the clinical treatment of secondary bacterial infection caused by H9N2 AIV could be provided.

## Materials and Methods

### Ethics Approval

The animal study protocol was approved by the South China Agricultural University Committee of Animal Experiments (approval ID, SYXK-2014-0136). The experiments were performed in accordance with the recommendations of the Guide for the Care and Use of Laboratory Animals of the National Institutes of Health.

### Animals and Viral Infection

One-day-old specific pathogen free (SPF) chickens and SPF chicken embryos were purchased from the SPF Experimental Animal Center of Guangdong Dahuanong Co., Ltd. The H9N2 SH01 strain was isolated and preserved in our laboratory. A total of 32 9-day-old healthy SPF chickens with a uniform body weight were selected and divided into four groups: The mock, H9N2 AIV infection (H9N2), AL feeding (AL), and H9N2 AIV infection with feeding AL (H9N2-AL) groups. H9N2 AIV infection was carried out by nasal drip and the infection dose of the H9N2 virus was 3-fold 10^6^TCID_50_/0.1 mL, and AL was administered at the same time as the viral infection, at a dose of 2.5 mL/kg/day for 3 days, and then put into a negative pressure isolator for routine feeding. At the same time of viral infection, 0.1 mL phosphate buffer saline (PBS) was dripped into nasal cavity of chickens in mock group and AL group.

### Detection of Virus Shedding

The cloacal swab was frozen and thawed repeatedly 3 times, and the virus RNA in the sample was extracted, according to the instructions of the AxyPrep humoral virus DNA/RNA small extraction kit (Axygen, CA, United States). A pair of identification primers were designed according to the HA gene of the H9N2 virus; the upstream sequence was 5′-CAAGATGGAAGTAGTATCACT-3′ and the downstream sequence was 5′-TTGCCAATTATATACAAATGT-3′. The extracted RNA was used as a template for the preparation of a one-step reverse transcription system and detected by RT-qPCR, as previous reported (Su et al., 2019).

### Detection of Viral Load by RT-qPCR

The tissue samples (lung and ileum) of the same quality were collected, and the total RNA in the tissue was extracted by TRIzol (Invitrogen, Carlsbad, CA, United States) reagent method, according to the manufacturer’s instructions. The total RNA of the obtained tissue sample was reverse transcribed into cDNA, according to the instructions of the genome removal reverse transcription kit (TiANGEN, Beijing, China). Detection of the viral copy number in each tissue was assessed by quantitative RT-PCR (RT-qPCR). The primers used for constructing standard curves were as follows: 5′-ACTCGATGAGCATGACGCAA-3′ and 5’-GGTCCCGTTCCGAATTGTCT-3’. RT-qPCR quantification of the viral load of each organ was conducted using standard curves, as previously described (Zhang et al., 2015).

### 16s rRNA High Throughput Sequencing of Intestinal Microbiota and Bioinformatics Analysis

The DNA of the ileum contents was extracted using a genomic DNA extraction kit. The V3 and V4 regions of bacterial 16S rDNA were amplified by RT-qPCR. The primers used were as follows: 5′-CCTACGGRRBGCASCAGKVRVGAAT-3′ and 5′-GGACTACNVGGGTWTCTAATCC-3′; a connector with Index was simultaneously added to the end of the fragment, and then a sequencing library was constructed. After the library was qualified, 2 × 300 bp double-terminal sequencing was carried out according to the instructions of the Illumina MiSeq instrument. The forward and backward reads obtained by sequencing were spliced, the splicing sequences containing N were filtered out and the sequences of > 200 bp were retained. The retained sequences were qualitatively filtered and the chimeric sequences deleted; the sequence operational taxonomic unit (OTU) cluster analysis was then performed using VSEARCH (1.9.6; with the similarity set to 97%). The representative sequence of OTU was then annotated and analyzed by RDP classifier Bayesian algorithm, and the species composition of each sample was calculated. Based on the results of OTU, the subsequent diversity difference analysis was carried out. Mothur (v.1.30) software was used for A index analysis (Schloss et al., 2009). Principal component analysis (PCA), principal coordinates analysis (PCoA), non-metric multidimensional scaling (NMDS) and unweighted pair group method with arithmetic mean (UPGMA) analysis was performed to analyze the diversity among different groups. The dominant bacterial community difference between groups was detected using Linear discriminant analysis effect size (LEfSe). LEfSe analysis was performed to analyze the significant differences between groups.

### Detection of Gene Expression in Ileal Tissue by RT-qPCR

The cDNA templates of ileal tissue samples were prepared, and RT-qPCR was used to detect gene expression. The PCR system used 20 μL volume according to the instructions of the kit (Roche Diagnostics, Montreal, QC, Canada), including 10 μL of 2 x SYBR Green qPCR Master Mix, 1 μL forward primer and 1 μL reversed primer, 1 μL RNA and 7 μL ddH_2_O; the primers of detecting the gene expression in ileal tissue were listed in Table 1. The cycling conditions were as follows: 30 cycles at 95°C for 10 min; 95°C for 10 s, 60°C for 30 s, 95°C for 15 s; 60°C for 1 min and 95°C for 15 s. At the end of the reaction, according to the CT value of the target gene and the internal reference gene, the relative expression of the target gene was calculated by the 2^–ΔΔ*CT*^ method (Zhang et al., 2019).

**TABLE 1:** Primers of detecting the gene expression in ileal tissue by RT-qPCR.

**Gene**	**Forward (5′-3′)**	**Reversed (5′-3′)**
*ZO-1*	GCCTGAATCAAACCCAGCAA	TATGCGGCGGTAAGGATGAT
*Claudin-3*	GAAGGGCTGTGGATGAACTG	GAGACGATGGTGATCTTGGC
*Occludin*	GATGGACAGCATCAACGACC	CATGCGCTTGATGTGGAAGA
*TNF-*α	TGTATGTGCAGCAACCCGTA	CCACACGACAGCCAAGTCAA
*IFN-*γ	ATCATACTGAGCCAGATTGTTTCG	TCTTTCACCTTCTTCACGCCAT
*iNOS*	AGTGGTATGCTCTGCCTGCT	CCAGTCCCATTCTTCTTCC
β*-actin*	CTGGCACCTAGCACAATGAA	CTGCTTGCTGATCCACATCT

### Red/ET Engineering-Related Strains and Plasmids

The *E. coli* Nissle1917 (EcN) strain, *E. coli* NeonGreen strain [GB05, ampicillin (amp) resistance modified by the NeonGreen fluorescence labeling gene], pSC101-BAD-gbaA-amp plasmid (pSC101 vector, amp resistance, red γβαA under PBAD promoter), pSC101-BAD-cre-tet plasmid [pSC101 vector, tetracycline (tet) resistance, Cre under PBAD promoter] and pR6K-lox71-cm-lox66 plasmid were all donated by Professor Zhang Youming (German College of Shandong University, Qingdao, Shandong).

### Tissue Bacteria Isolation

The aseptic isolated liver, lung and mesentery were homogenized. Homogenate (100 μL) was mixed on a Luria broth (LB) plate, and the bacterial solution was coated and cultured in a 37°C incubator for 12 h. When the bacterial growth was observed on the plate, the colony growth was judged to be positive, while the non-colony growth was judged to be negative.

### NeonGreen-Tagged Bacteria Isolation and Quantification

A total of 36 9-day-old healthy SPF chickens with a uniform body weight were purchased and divided into three groups: The mock, H9N2 AIV infection (H9N2) and H9N2 AIV infection with feeding AL (H9N2-AL) groups. At the age of 9 days, the infection dose of the H9N2 virus was 3-fold 10^6^ TCID_50_/0.1 mL, and AL was fed to the chickens at the same time as the viral infection at a dose of 2.5 mL/kg/day, which lasted 3 days. On day 3 after viral infection, all chickens were fed NeonGreen bacteria at a dose of 300 mL 1 × 10^9^ CFU/mL per chicken, which was routinely raised in a negative pressure isolator.

Samples were taken at 12, 24, 36 and 48 h after the administration of NeonGreen bacteria. The mixture containing ileum, liver, lung and mesentery inner wall and lumen tissues was aseptically collected and homogenized in a sterile homogenate tube filled with 1 mL saline. Homogenate mixture (100 μL) was applied to the LB&Amp plate, and the bacterial solution was coated in a 37°C incubator for 12 h. When the bacterial growth on the plate was observed, the colony growth was judged to be positive, while the non-colony growth was judged to be negative.

RT-qPCR was performed for the count of NeonGreen *E. coli* bacteria. The total volume of DNA extracted was derived from enteric contents, mesentery, lung and liver. The DNA was adjusted to the same concentrations. DNA standards were prepared from *E. coli* strains carrying plasmids with *E. coli* fragment inserts, which was isolated from a poultry farm (GenBank No. MG602206). The abundance of the gene was evaluated by multiplying the number of copies per well by the total volume of DNA per well. The total reaction volume of 20 μL contained 1.0 μL DNA, 10.0 μL SYBR Green qPCR Mix (Roche Diagnostics, Shanghai, China), 0.5 μL of each primer and 8 μL H_2_O. Samples were amplified by the following conditions: Initial denaturation at 94°C for 2 min, 40 cycles of heat denaturation at 94°C for 10 s, primer annealing at 60°C for 40 s. Fluorescence signal acquisition was set at 60°C. The primers for *E. coli* amplification were as follows: 5′-GTTAATACCTTTGCTCATTGA-3′ and 5′-ACCAGGGTATCTTAATCCTGTT-3′.

### Metabonomic Analysis of Intestinal Contents

The collected ileum contents were sent to Shanghai Matt painting Biotechnology Co., Ltd., for non-targeted metabonomics. Gas chromatography time-of-flight mass spectrometry based on silanization derivation was used as the analytical chromatographic mass. A metabolite database, JIALIB, was established through the identification of high-purity chemical standards of metabolites. The statistical analysis of the data was performed based on conventional multivariate statistical methods, such as PCA, Partial Least Square Discriminant Analysis (PLS-DA) and Orthogonal Projections to Latent Structures Discriminant Analysis (OPLS-DA). The metabolic pathway enrichment analysis used a specific hypergeometric algorithm to enrich the information of metabolites, weigh their contribution and identify key metabolic networks.

### Construction of EcN Amp Resistant Strain (EcN:Amp)

In order to screen and count the strains in the later stage, the resistant high-copy plasmid was transformed into the target strain to carry the corresponding resistance function. In the present study, the pMD-19T (Control Insert) plasmid was electrotransformed to make EcN carry Amp resistance. The specific transfer steps of EcN were conducted as previously described (Bian et al., 2012).

### Construction of EcN Mutants (ΔmoaA and ΔAZG)

In order to construct nitrate metabolism deletion strains, molybdenum cofactor genes moaA and nitrate reductase genes *napA*, *narZ* and *narG* in the nitrate metabolism were deleted (Winter et al., 2013), the target genes were replaced by lox71-cm-lox66 fragments by RedE/T recombination engineering (Wang et al., 2016), the resistant fragments between lox sites were then deleted by a site-specific recombination system, and the target genes were repeatedly knocked out using this strategy to obtain gene deletion strains. In this experiment, Δ*moaA* mutant only replaced the *moaA* gene with the cm resistance gene, while the Δ*AZG* mutant in turn knocked out the *napA* and *narZ* genes, and finally replaced the *narG* gene with the chloramphenicol resistance gene. The specific steps of constructing mutants were conducted as previously described (Autieri et al., 2007; Liu et al., 2016). The primer sequences for target gene replacement were designed based on the lox site of the pR6K-lox71-cm-lox66 plasmid sequence and the target knockout gene sequences were shown in Table 2. According to the position of the homologous arm of the linear substrate, identification primers were designed at both ends of the homologous arm of the target knockout gene in EcN to identify the sequence changes before and after substitution and knockout by RT-qPCR. The primer sequences of the identification of recombinants by RT-qPCR were shown in Table 3.

**TABLE 2:** Primer sequence of target gene replacement.

**Primers**	**Sequence(5′-3′)**
*moaA* del-F	atggcttcacaactgactgatgcatttgcgcgtaagttttactacttgcgGCAAGGGCTGCTAAAGGAAGCG
*moaA* del-R	gcaggcggttgcaagtggcgcagaagtctttttcatacggcatgataaggGGGCAGGATAGGTGAAGTAGG
*napA* del-F	atgaaactcagtcgtcgtagctttatgaaagctaacgccgttgcggccgcGCAAGGGCTGCTAAAGGAAGCG
*napA* del-R	tgtcgccacggcgcagatcgcgcgctttcgcatccagcgggtgaataaacGGGCAGGATAGGTGAAGTAGG
*narZ* del-F	atgagtaaacttttggatcgctttcgctacttcaaacaaaagggcgaaacGCAAGGGCTGCTAAAGGAAGCG
*narZ* del-R	cgcgcatgccagttacttccgaaccaggaatattcataatgcgttcctggGGGCAGGATAGGTGAAGTAGG
*narG* del-F	atgagtaaattcctggaccggtttcgctacttcaaacagaagggtgaaacGCAAGGGCTGCTAAAGGAAGCG
*narG* del-R	cacgctgttgggtaatttccgaaccaggcaggttaacgatacgttcctgcGGGCAGGATAGGTGAAGTAGG

*Lowercase letters are homology arms of each gene.*

**TABLE 3:** Primer sequences of the identification for the recombinants by PCR.

**Primers**	**Sequence (5′-3′)**
*moaA* check -F	CTCCCGTATCTGGAAAGGTG
*moaA* check-R	GATGGAGTTTACCAATGGAG
*napA* check-F	ACCAGCAGGAAGAGCAAGGTG
*napA* check-R	AGATCACTTCGCCACGGCGAG
*narZ* check-F	TTCCTGGAGCAGGAGTTATG
*narZ* check-R	GCGTTGGTTTCGGGCAAACG
*narG* check-F	TAGCAATGTCGATTTATCAG
*narG* check-R	TACGGGTGACCGAGTTATGG

### *In vitro* Competitive Testing of Mutant Strains

EcN:Amp and EcN strain Δ*moaA* were simultaneously added to the cell culture flask containing 10 mL LB liquid medium at 1:1, and their final concentration was 1 × 10^4^ CFU/mL. At the same time, KNO3 (100 μM) was added to the culture system in the treatment group, repeated 3 times in each treatment group, sealed in an anaerobic incubator and transferred to a 37°C incubator for culture. After 24 h, the number of EcN:Amp and EcN Δ*moaA* strains in each culture bottle was counted. The viable bacterial count of 3 mutant strains (EcN:Amp, EcN Δ*moaA* and EcN Δ*AZG*) was determined, the bacterial liquid was diluted 10 times, the sample homogenate with suitable 3-5 continuous dilutions was selected, and the 1 mL was poured into the plate containing antibiotics ampicillin and chloramphenicol; 2 plates were made for each dilution, and the dilution was compared at the same time. Following incubation at 37°C for 18–24 h, the plate colony count was determined, and the plate record of colony numbers between 15–150 CFU was selected. Finally, the competitive index (CI) of EcN:Amp and EcN strain Δ*moaA* with or without nitrate treatment was calculated. The competitive culture of EcN:Amp and EcN Δ*AZG* was carried out according to the above steps, and the CI of EcN:Amp and EcN strain Δ*AZG* was calculated with or without nitrate treatment.

### Verification of Nitrate Metabolic Function *in vivo*

A total of 16 9-day-old healthy SPF chickens with uniform body weight were divided into four groups: The mock, H9N2 AIV infection, feeding *S*-methyl-isothiourea (SMT) after H9N2 AIV infection, and feeding AL after H9N2 AIV infection groups. At the age of 9 days, the infection dose of the H9N2 virus was 3-fold 10^6^ TCID_50_/0.1 mL, and the mixed bacterial solution of EcN:Amp and EcN Δ*moaA* was administered at the same time as the viral infection at a dose of 100 μL/per chicken, 1 × 10^10^ CFU/mL; SMT and AL were fed to them on day 1 after viral infection. The dose of SMT was 10 mg/per/d and that of AL 2.5 mL/kg, lasting for 3 days, and put into the negative pressure isolator for routine feeding. The above animal experiments were repeated with EcN:Amp and EcN strain Δ*AZG*.

On day 5 after viral infection, the ileum tissue near the ileocecal junction was cut and sampled. Following rinsing with phosphate-buffered saline (PBS), the intestinal tissue was lysed using cell and tissue lysate reagent, and the nitrate content in the intestinal tissue was detected, according to the instructions of the total nitric oxide detection kit. In addition, the contents at the junction of the blind were collected in the aseptic environment and, following weighing, each sample was diluted with PBS, according to a fixed mass-volume ratio. The sample was then used as the original solution. Next, the number of bacteria was identified and the competition index was calculated.

## Results

### Determination of Tissue Viral Load in SPF Chickens Following H9N2 AIV Infection and AL Treatment

**FIGURE 1:**
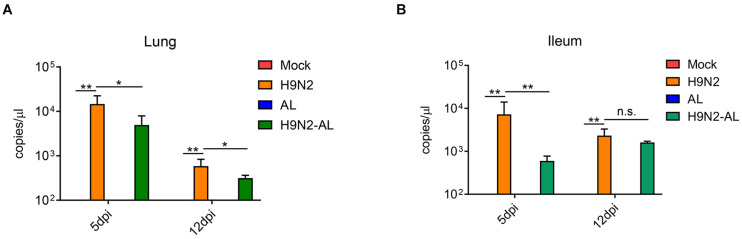
Determination of viral load in lung and ileum tissue 5 and 12 days after H9N2 AIV infection in SPF chickens by RT-qPCR. **(A)** Viral load in the lung 5 and 12 days after H9N2 AIV infection in chickens. **(B)** Viral load in the ileum 5 and 12 days after H9N2 AIV infection in chickens. Data are presented as the mean ± standard deviation (*n* = 3). The differences between groups were analyzed using ANOVA. *P < 0.05, **P < 0.01 and n.s. represents not significant; dpi, day post infection; AL, ageratum-liquid.

Cloacal swabs were collected on day 3 after viral infection for the RT-PCR detection of the HA gene of H9N2 AIV. Results showed that the cloacal swabs collected from SPF chickens infected with the virus can be amplified into HA fragments of H9N2 AIV, indicating that SPF chickens were infected successfully in this experiment (Supplementary Figure 1). Lung and ileum tissues were collected on days 5 and 12 after H9N2 AIV infection, and the viral load was determined by RT-qPCR. The results showed that the virus could be detected in the lungs and ileum at both time points, indicating that the influenza virus could proliferate effectively in SPF chickens and intestinal epithelial cells (*P* < 0.01, Figure 1). The feeding of AL 5 days after viral infection in SPF chickens could significantly reduce the viral load in the lung and ileum epithelium (*P* < 0.01 and *P* < 0.05, Figure 1), indicating that AL might exert its antiviral effect by affecting intestinal function.

### α Diversity Index Statistics and β Diversity Analysis of Gut Microbiota Following H9N2 AIV Infection and AL Treatment in SPF Chickens

α diversity reflects the species richness and diversity of a single sample (Mancini et al., 2018). The Chao and Ace index indicate species richness (Xiao Joe et al., 2019). As shown in Supplementary Figures 2A,B, there was no significant change in community species richness on the fifth day after H9N2 AIV infection (*P* > 0.05), indicating that H9N2 AIV infection did not cause the change of intestinal community species richness. The species richness of the AL group increased significantly compared with the mock group (*P* < 0.05), and that of the H9N2-AL group increased significantly compared to the H9N2 AIV infection group, indicating that AL could increase the intestinal community species richness (Supplementary Figures 2A,B). Shannon and Simpson index measure species diversity; the higher the Shannon index value, the higher the species diversity, while the opposite is true for the Simpson index (Kim et al., 2017). As shown in Supplementary Figures 2C,D, the species diversity of the intestinal flora decreased significantly on day 5 in the H9N2 AIV infection group, while the species diversity of the intestinal flora in the AL and H9N2-AL groups increased significantly compared to the mock and H9N2 infection groups (*P* < 0.01), indicating that H9N2 AIV infection can destroy intestinal flora diversity, while AL can restore it. OTU analysis-based dilution and grade abundance curves in different groups were shown in Supplementary Figures 2E,F. As compared with the mock and H9N2 AIV infected groups, the sample curve of the group fed with AL and the group fed with AL following infection was broader on the horizontal axis, indicating that the species composition of the AL and H9N2-AL groups was relatively rich and the species composition was more specific compared with mock and H9N2 AIV infected groups.

β diversity analysis, which included PCA, PCoA, NMDS and UPGMA analysis, showed that the samples of the same treatment group were concentrated in one area, while the sample distances of different groups were concentrated in different areas, with marked differences between the mock and H9N2 AIV infection groups at the 5 day time point, which indicated that the similarity of samples between the mock and H9N2 AIV infection groups was low. By contrast, the sample distance between the single feeding AL and H9N2 AIV infection with feeding AL groups were close to each other, indicating a high species similarity between the two groups (Supplementary Figures 2G–I). According to the distance, the trend of the four groups of samples was divided into 3 parts that represent the effects of different treatments on the similarity of intestinal flora composition in chickens, indicating that H9N2 AIV infection caused changes in intestinal diversity and AL could also improve intestinal flora diversity (Supplementary Figure 2J).

### Taxonomic Analysis of Gut Microbiota Following H9N2 AIV Infection and AL Treatment in SPF Chickens

**FIGURE 2:**
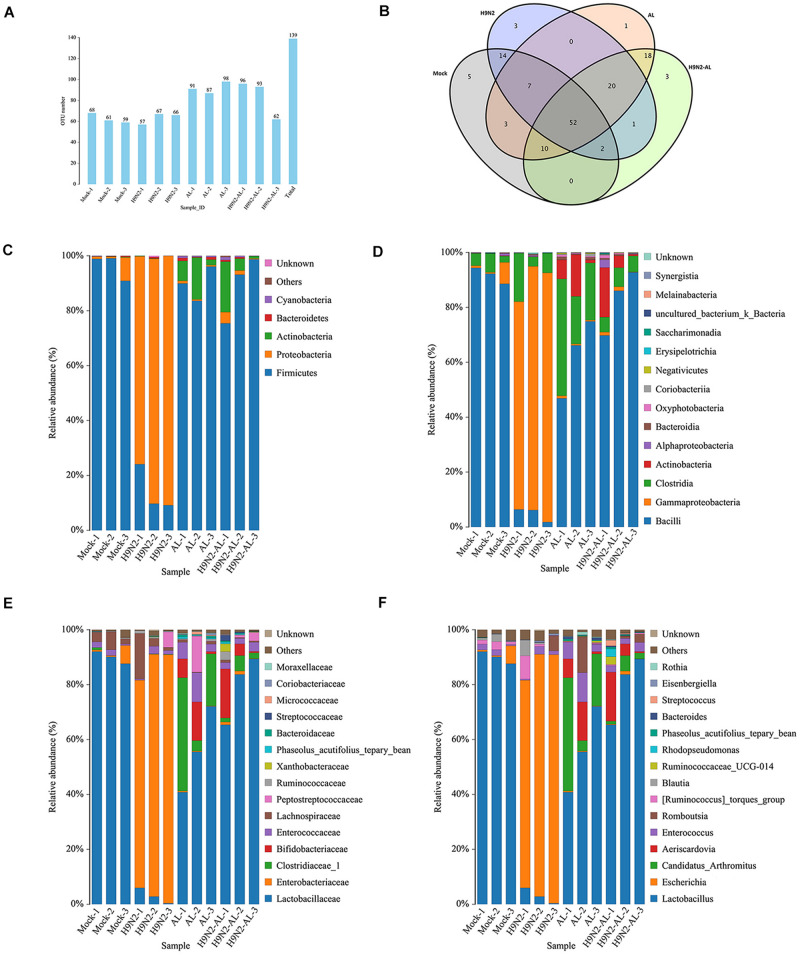
Taxonomic analysis of gut microbiota 5 days after H9N2 AIV infection and AL treatment in SPF chickens. **(A)** Distribution map of OTU number in samples from different treatment groups. **(B)** Venn diagrams of OTU in different treatment groups. Relative abundance of intestinal microflora at the **(C)** phylum, **(D)** class, **(E)** family and **(F)** genus levels in different treatment groups. AL, ageratum-liquid; OTU, operational taxonomic unit (OTU).

According to the sequencing results, sequences with a similarity of > 97% were defined as an OTU, with each OTU corresponding to a representative sequence. As shown in Figure 2A, the number of OTUs of each sample in each group was almost the same, with a total of 139 OTUs in 12 samples. A Venn map of the four groups showed that there were 5, 3, 1 and 3 unique OTUs in the mock, H9N2 AIV infection, AL feeding and H9N2 AIV infection with feeding AL groups, respectively, at 5 days post infection, indicating that different treatments can cause changes in the intestinal flora and produce corresponding unique flora (Figure 2B).

By comparing the OTU sequences with the reference database, the composition of the flora at each classification level was obtained. As shown in Figure 2C, at the phylum level, the dominant flora in the mock group was *Firmicutes*, with an average relative abundance of 96%. Secondly, the relative abundance of *Proteobacteria* reached 3%. Following infection with H9N2 AIV, the relative abundance of *Proteobacteria* became the dominant flora, with an average relative abundance of 85%, while that of *Firmicutes* decreased to 14%, indicating that H9N2 AIV could cause intestinal flora disorder and lead to the proliferation of *Proteobacteria*. In the AL group, the average relative abundance of the phylum *Firmicutes* accounted for 89%, followed by *Actinobacteria*, with an average relative abundance of 8%. In the H9N2-AL group, the phylum *Firmicutes* returned to the dominant flora, with an average relative abundance of 89%, followed by *Actinobacteria* with an average of 8%, and *Proteobacteria* with an average of 2%. The above results suggested that H9N2 AIV can induce intestinal flora disorder and cause a high proliferation of *Proteobacteria*, while AL can improve the intestinal microecological environment, inhibit the proliferation of *Proteobacteria* and promote the proliferation of *Firmicutes* and *Actinobacteria* into the dominant flora.

In the mock group, at the class, family and genus levels, the first dominant flora was *Bacilli*, *Lactobacillaceae* and *Lactobacillus*, respectively. The second dominant flora was *Gamma*proteobacteria, *Enterobacteriaceae* and *Escherichia*. The average relative abundance of *Lactobacillus* and *Escherichia* in the mock group was 90 and 2.5%, respectively (Figures 2D–F). On day 5 following H9N2AIV infection, the relative abundance of *Lactobacillus* decreased to 3%, while that of *Escherichia* increased to 85% (Figure 2F). In the AL group, at the genus level, the main flora was *Lactobacillus*, *Candidatus-Arthromitus*, *Aeriscardovia*, *Enterococcus* and *Romboutsia*, with their relative abundance at 56, 21, 7, 6 and 4%, respectively (Figure 2F). In the H9N2-AL group, the main flora was *Lactobacillus*, *Aeriscardovia*, *Candidatus-Arthromitus* and *Enterococcus*, with a relative abundance of 79, 7.5, 3 and 2.5% at the genus level, respectively (Figure 2F).

In combination, H9N2 AIV infection can destroy intestinal homeostasis, causing an increase in intestinal opportunistic pathogens, like *Escherichia* and inhibiting the growth of beneficial bacteria, like *Lactobacillus*. AL can improve intestinal homeostasis, restore the micro-ecological environment destroyed by H9N2 AIV, and lead to *Lactobacillus* becoming the dominant flora again.

### LefSe Analysis of Gut Microbiota Following H9N2 AIV Infection and AL Treatment in SPF Chickens

**FIGURE 3:**
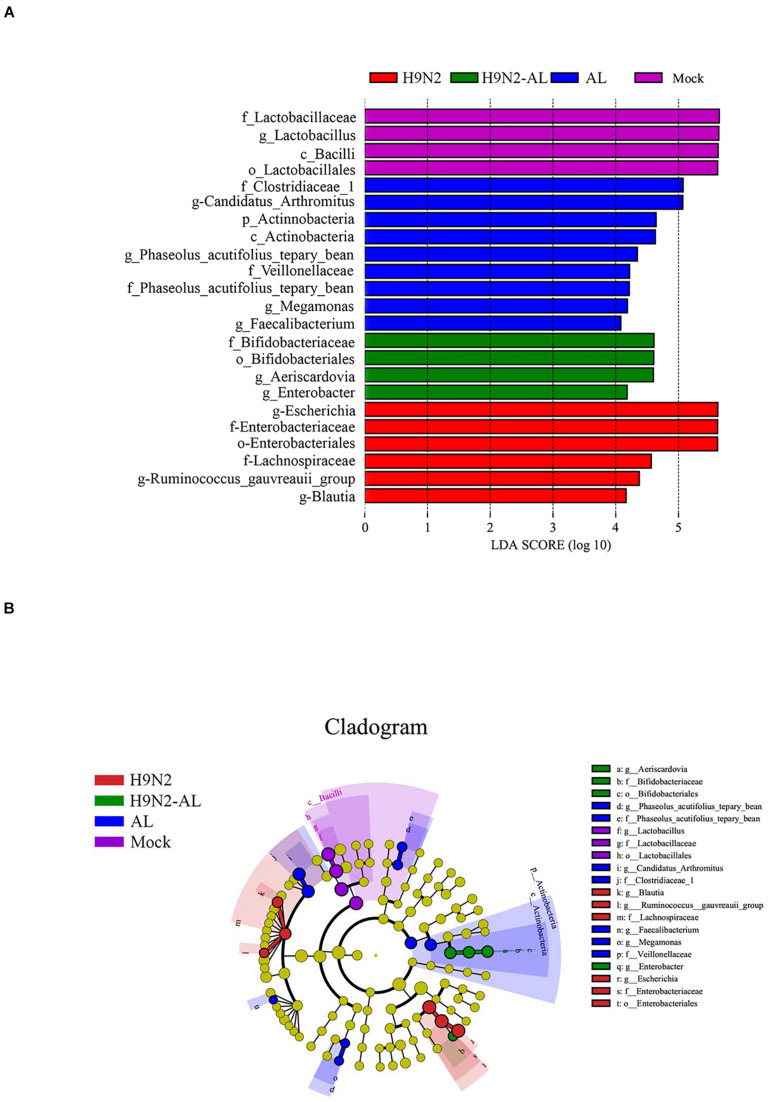
LefSe analysis of gut microbiota at 5 days after H9N2 AIV infection and AL treatment in SPF chickens. **(A)** Histogram of LDA value distribution in different treatment groups. **(B)** LEfSe analysis of evolutionary branching diagram in different treatment groups. AL, ageratum-liquid; LDA, linear discriminant analysis; LefSe, LDA effect size.

In order to further screen the biomarker in the changes of intestinal flora caused by different treatments, LefSe analysis was performed. With a linear discriminant analysis (LDA) score of > 4.0 as the threshold, the species were screened from the phylum to the genus level. As shown in Figure 3A, the biomarkers used for the mock group were *Bacilli*, *Lactobacillaceae*, *Lactobacillus* and *Lactobacillales*. The biomarkers used for the H9N2 AIV infection group on day 5 were *Escherichia*, *Enterobacteriaceae*, *Enterobacteriales*, *Lachnospiraceae*, *Ruminococcus-gauvreauii* and *Blautia*. The biomarkers used for the AL group were *Clostridiaceae*, *Candidatus-Arthromitus*, *Actinobacteria*, *Phaseolus acutifolius* (tepary beean), *Veillonellaceae*, *Phaseolus acutifolius* (tepary bean), *Megamonas* and *Faecalibacterium*. The biomarkers used for the H9N2-AL group were *Bifidobacteriaceae*, *Bifidobacteriales*, *Aeriscardovia* and *Enterobacter* on day 5 post infection. According to the biological classification of bacteria and LDA score, the biomarker for each treatment group was further screened. *Lactobacillus*, *Escherichia*, *Candidatus-Arthromitus* and *Aeriscardovia* were the biomarkers used for the mock, H9, AL and H9N2-AL groups, respectively.

In order to more intuitively judge the changes in the microflora caused by different treatments, an evolutionary branching tree was made, and the circles radiated from inside to outside, representing the taxonomic level from the phylum to the genus, respectively. As shown in Figure 3B, the species with statistical differences at > 3 consecutive classification levels were *Lactobacillus*, *Escherichia* and *Aeriscardovia*. In combination, H9N2 AIV infection caused intestinal flora disorder, and the possible bacterial biomarkers for the mock and infection groups were *Lactobacillus* and *Escherichia*, respectively. After feeding the infection group with AL, the biomarker was *Aeriscardovia*.

### Effects of H9N2 AIV Infection and AL Treatment on Tight Junction of Ileum Epithelium of SPF Chickens

**FIGURE 4:**
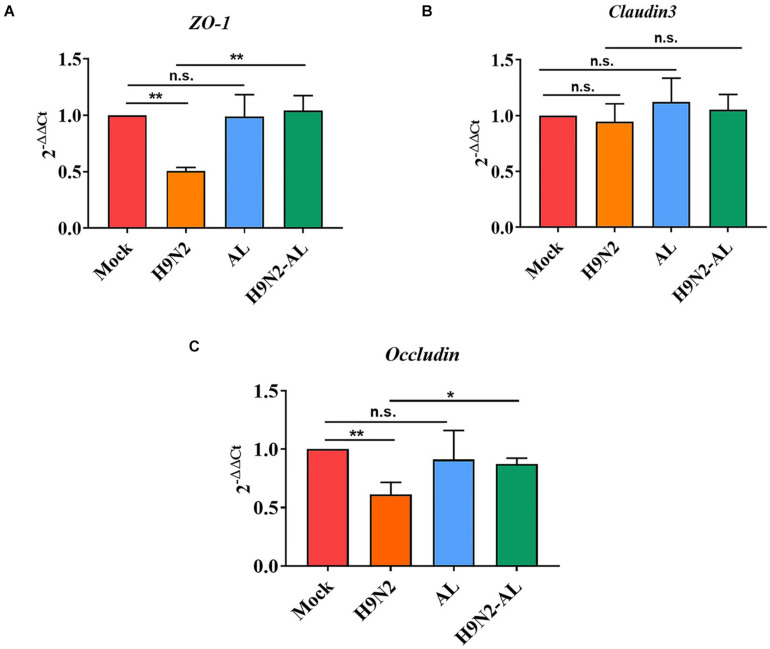
Effect of H9N2 AIV infection and AL treatment on mRNA expression of tight junction protein in the ileum epithelium of SPF chicken 5 days post-infection. Detection of mRNA expression of tight junction proteins **(A)** *ZO-1*, **(B)** *Claudin 3* and **(C)** *Occludin* in ileum epithelial cells by RT-qPCR. Data are presented as the mean ± standard deviation (*n* = 3). The differences between groups were analyzed using ANOVA. *P < 0.05 and **P < 0.01. n.s., not significant. AL, ageratum-liquid.

In order to explore the barrier function of H9N2 AIV on intestinal mucosal epithelial cells, the mRNA expression of scaffold protein *ZO-1*, and transmembrane proteins *Claudin 3* and *Occludin* was measured. As shown in Figure 4, the mRNA expression level of genes *ZO-1* and *Occludin* was significantly decreased (5 dpi, *P* < 0.01), while that of *Claudin* 3 did not change significantly (*P* > 0.05), indicating that H9N2 AIV infection can cause the destruction of tight junction proteins in intestinal epithelial cells. There was no significant difference in the expression of intestinal epithelial tight junction proteins between the mock and AL groups (*P* > 0.05), while AL treatment in the infection group could upregulate the *ZO-1* and *Occludin*, as compared with the infection group (*P* < 0.01 and *P* < 0.05), indicating that AL can protect the structure and function of the intestinal epithelial mucosa to a certain extent.

### Effects of H9N2 AIV Infection and AL Treatment on Intestinal Bacterial Translocation in SPF Chickens

H9N2 AIV infection is often accompanied by secondary bacterial infection in visceral organs. The present results showed that bacteria were isolated from the lungs and mesentery at 5 dpi and were positive in all tissues at 12 dpi, while in the H9N2-AL group, bacteria were only isolated from the lungs at 5 dpi (Table 4). These findings suggested that H9N2 AIV infection can cause bacterial secondary infection in tissues and organs and that AL can prevent infection.

**TABLE 4:** Isolation of bacteria in different tissues and organs of different treatment groups.

**Days post infection**	**Position**	**Mock**	**H9N2**	**AL**	**H9N2-AL**
5 dpi	Liver	−(3/3)	−(3/3)	−(3/3)	−(3/3)
	Lung	−(3/3)	**+ (2/3)**	−(3/3)	**+ (1/3)**
	Mesentery	−(3/3)	**+ (2/3)**	−(3/3)	−(3/3)
12 dpi	Liver	−(3/3)	**+ (1/3)**	−(3/3)	−(3/3)
	Lung	−(3/3)	**+ (2/3)**	−(3/3)	−(3/3)
	Mesentery	−(3/3)	**+ (2/3)**	−(3/3)	−(3/3)

*+ means isolated bacteria was positive; Bold number (front) /Bold number (back): bold number (front) means the number of positive isolated bacteria; bold number (back) means the number of total isolated bacteria.*

Following H9N2 AIV infection, bacteria can be isolated in the mesentery, lung and liver, indicating that there is a phenomenon of bacterial translocation (Table 4). Our previous results also showed that H9N2 AIV infection can cause the destruction of intestinal barrier function (Figure 4). It was therefore speculated that H9N2 AIV infection causes intestinal homeostasis disorder and intestinal opportunistic bacterial translocation, leading to bacterial secondary infection. To further test this speculation, the fluorescence-labeled bacterial strain NeonGreen was used to trace bacterial translocation. Results showed that NeonGreen-tagged bacteria were isolated from the intestinal cavity, indicating that the flora entered the intestine. In the H9N2 AIV infection group, NeonGreen-tagged bacteria was positive in the mesentery and lung at 24-48 h, and in the liver at 36-48 h, indicating that the H9N2 AIV infection promoted bacterial translocation to the mesentery and other tissues through the intestinal wall. Bacteria were isolated only in the lungs at 36 h in the H9N2-AL group, indicating that AL could effectively inhibit bacterial translocation through the intestinal wall to mesentery, lung and liver (Supplementary Table 1). Furthermore, the NeonGreen-tagged *E. coli* bacteria count in different treatment groups was shown in Supplementary Figure 3. Results showed that the count of NeonGreen-tagged *E. coli* from intestinal cavity in H9N2-NeonGreen group was significantly higher than that in NeonGreen group and H9N2-AL-NeonGreen group at 12 h, 24 h, 36 h and 48 h. The count of NeonGreen-tagged *E. coli* from mesentery in H9N2-NeonGreen group was gradually increasing from 24 h to 48 h, however, no NeonGreen-tagged *E. coli* was detected in NeonGreen and H9N2-AL-NeonGreen groups. Besides, the count of NeonGreen-tagged *E. coli* was gradually increasing from 24 h to 48 h in lung, however, no NeonGreen-tagged *E. coli* was detected in NeonGreen and H9N2-AL- NeonGreen groups at 12 h, 24 h and 48h, although the NeonGreen-tagged *E. coli* was detected in H9N2-AL-NeonGreen group at 36 h, while the count was significantly lower than that in the H9N2-NeonGreen group. Similarly, the count of NeonGreen-tagged *E. coli* in liver was gradually higher from 36 h to 48 h in H9N2-NeonGreen group, but no NeonGreen-tagged *E. col*i was detected in NeonGreen group and H9N2-AL- NeonGreen group from 12 h to 48 h, illustrating that H9N2 AIV infection causes NeonGreen-tagged *E. col*i translocation, leading to bacterial secondary infection, while AL could effectively inhibit *E. col*i translocation through the intestinal wall to mesentery, lung and liver.

In combination, H9N2 AIV infection promotes bacterial translocation from the intestinal cavity to the mesentery, lung and liver and causes secondary bacterial infection. AL can effectively prevent bacterial translocation induced by H9N2 AIV.

### Effects of H9N2 AIV Infection and AL Treatment on Intestinal Metabolism of SPF Chickens

A multivariate model was used to construct a multivariate control chart to evaluate the validity of each sample data. Results showed that the biological variability of metabolic group samples was small and could be used for follow-up data analysis (Supplementary Figure 4A). By comparing the data of metabolic substances obtained by sequencing with the database of standard metabolic substances, 154 metabolic substances, 45 metabolic ratios and 211 unknown substances were obtained (Supplementary Figure 4B). The 154 substances annotated were chemically classified as follows: Amino acids (31%), carbohydrates (20%), organic acids (14%), nucleotides (9%), fatty acids (7%), lipids (6%), alkylamines (4%), alcohols (3%) and others (6%) (Supplementary Figure 4C). The 3D-PCA and 3D-PLS-DA showed that the sample clustering trends between the mock and H9N2 infection groups, or between H9N2 infection and H9N2-AL groups were different (Supplementary Figures 4D,E). In combination, both H9N2 AIV and AL can cause changes in the intestinal metabolic spectrum. OPLS-DA and VIP index volcano maps were constructed to analyze the changes in the metabolic spectrum between the mock and H9N2 AIV infection groups, and between the H9N2 AIV infection and H9N2-AL groups. Visualization of overall metabolite profile differences between the mock and H9N2 AIV infection groups or between the H9N2 and H9N2-AL groups were illustrated in Supplementary Figures 4F–I. Total 11 differential metabolites were screened out in the H9N2 AIV infection group, as compared with the mock group (Table 5). Total 5 differential metabolites were screened out in the H9N2-AL group, as compared with the H9N2 group (Supplementary Table 2).

**TABLE 5:** The differential metabolites were screened out in the H9N2 AIV infection group, as compared with the mock group (log2FC > 1.5, *P*-Value < 0.05, VIP > 1).

**Name**	**VIP**	**Corr**	***P* Value**
Putrescine	1.7354	0.5691	0.0459
Spermidine	2.6496	0.7861	0.0011
L-Alanine	2.2863	0.6789	0.0110
Sarcosine	2.5407	0.7711	0.0017
3-Aminoisobutanoic acid	2.2132	0.6311	0.0221
Ratio of Sarcosine/Dimethylglycine	1.9114	0.5999	0.0325
Ratio of Sarcosine/Glycine	2.2897	0.6308	0.0221
Ratio of L-Glutamic acid/L-Glutamine	2.2009	0.6456	0.0181
D-Mannose	2.5342	−0.7172	0.0056
Xanthosine	2.0657	−0.5923	0.0356
2-Hydroxy-3-methylbutyric acid	1.9631	0.6368	0.0204

**FIGURE 5:**
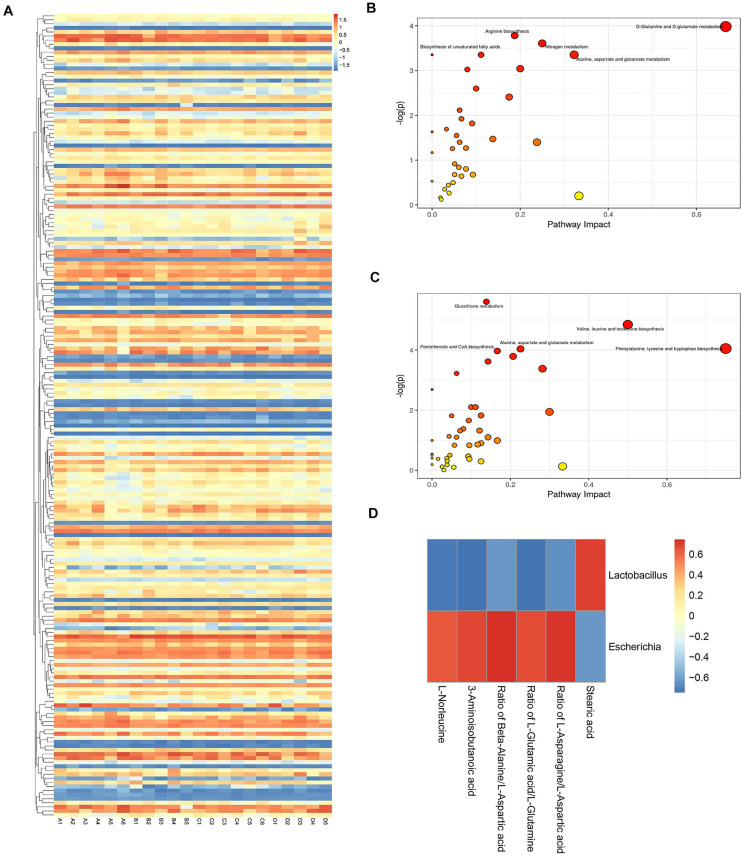
Metabolite profile visualization, metabolic pathway enrichment analysis and analysis of the correlation between intestinal flora and metabolic changes. **(A)** Heatmap depicting the overall metabolic variations among different treatment groups. **(B)** Differential metabolic pathway between the mock and H9N2 AIV infection groups. **(C)** Differential metabolic pathway between H9N2 AIV infection and H9N2-AL groups. **(D)** Corrplot of association analysis of microflora and metabolites. The number of sequencing samples in each group was ≥ 5. **(A)**, mock group; **(B)**, H9N2 AIV infection group; **(C)**, AL feeding group; **(D)**, after H9N2 infection group with feeding AL group.

The heatmap provides an overview of the global metabolic profiles among all samples, revealing the relative variations of each individual metabolite across all study samples in different treatment groups (Figure 5A). Overview of pathway analysis between H9N2 AIV infection group and the mock group, or between H9N2-AL group and H9N2 AIV infection group were shown in Figures 5B,C. In order to further determine the direct relationship between intestinal bacteria and metabolic changes, bacterial biomarker *Lactobacillus* from the mock group and *Escherichia* from the H9N2 AIV infection group were selected and screened according to the absolute value of a correlation coefficient of > 0.5. A total of 6 metabolites were found to be associated with *Lactobacillus* and *Escherichia*, in which the L-Glutamic acid/L-Glutamine ratio was positively correlated with *Escherichia* (Figure 5D).

### Effects of H9N2 AIV Infection and AL Treatment on Inflammatory Gene Expression of *TNF-*α, *IFN-*γ and *iNOS* in the Intestinal Mucosa of SPF Chickens

**FIGURE 6:**
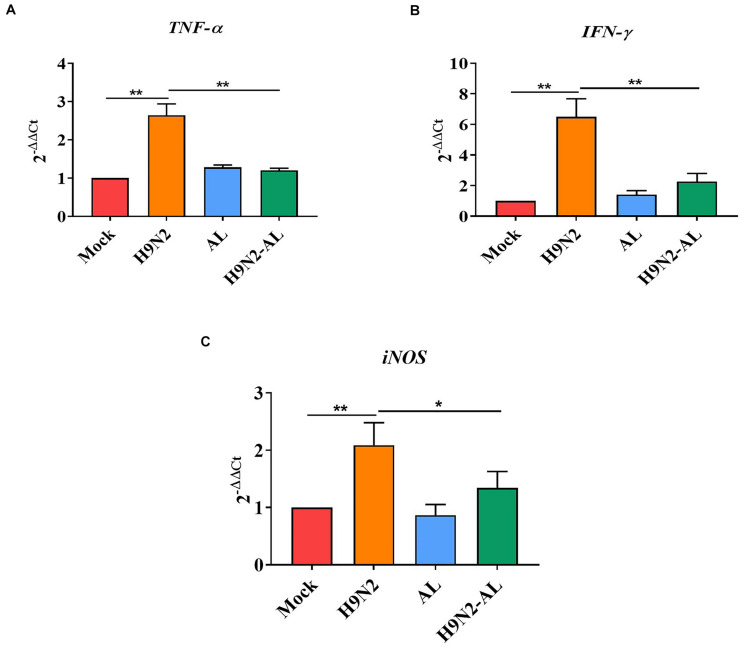
mRNA expression of pro-inflammatory genes in the intestinal mucosal epithelial cells of the ileum in different treatment groups. mRNA expression of **(A)** *TNF-*α, **(B)** *IFN-*γ and **(C)** *iNOS* in the intestinal mucosal epithelial cells of the ileum in different treatment groups 5 days post-H9N2 AIV infection. Data are presented as the mean ± standard deviation (*n* = 3). The differences between groups were analyzed using ANOVA. *P < 0.05 and **P < 0.01. AL, ageratum-liquid.

It was reported that iNOS is the sole source of nitric oxide during inflammation and TNF-α and IFN-γ are important inflammatory cytokines, and IFN-γ can induce the production of iNOS (Winter et al., 2013). The expression of *TNF-*α, *IFN-*γ and *iNOS* were therefore determined by RT-qPCR. The results showed that the gene expression of *TNF-*α and *IFN-*γ was significantly increased following H9N2 AIV infection (*P* < 0.01, Figures 6A,B). The gene expression of *iNOS* was also significantly increased following H9N2 AIV infection (*P* < 0.01; Figure 6C), while that of *TNF-*α, *IFN-*γ and *iNOS* was significantly downregulated in the H9N2 AIV infection group with AL feeding, as compared with the infection group (*P* < 0.01 and *P* < 0.05, Figures 6A–C). The results illustrated that H9N2 AIV infection increased the expression of inflammatory cytokines in intestinal epithelial cells, and that AL could inhibit the inflammation induced by the H9N2 AIV infection.

### Construction of EcN Mutant and *in vitro* Competitive Tests of Mutant Strains

**FIGURE 7:**
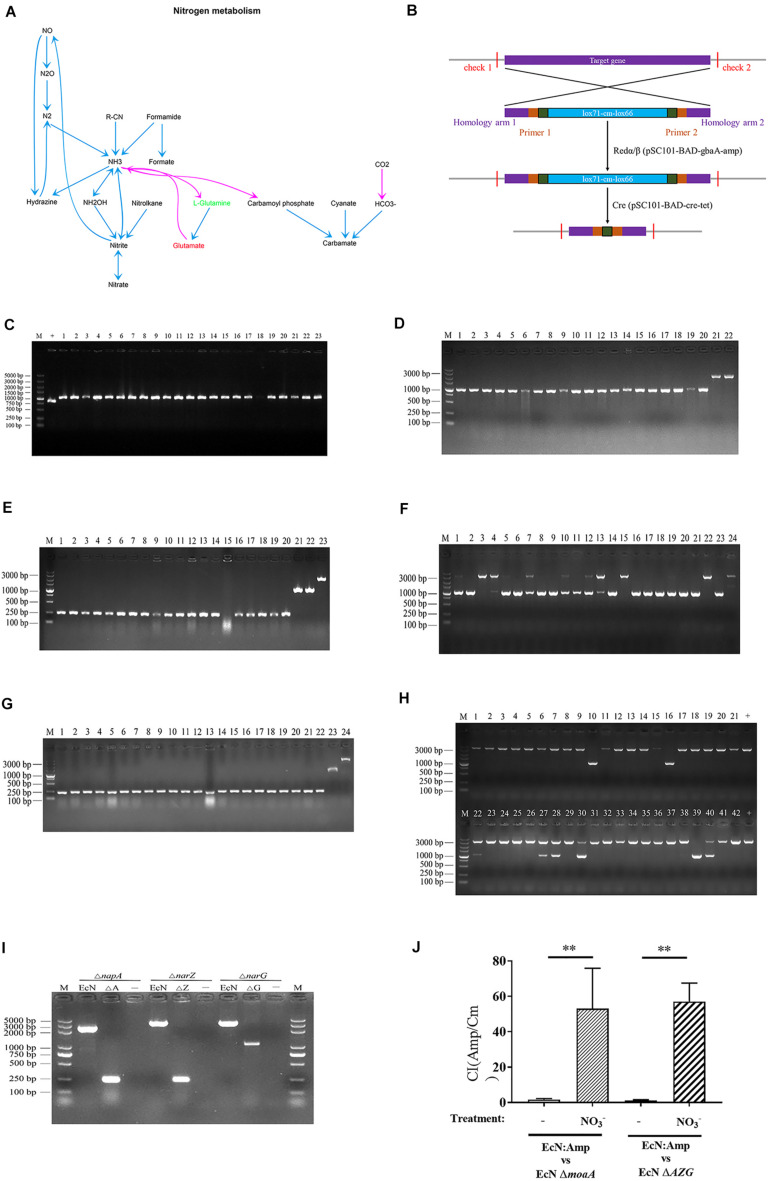
Construction of *EcN* mutant and *in vitro* competitive testing of mutant strains. **(A)** Metabolic pathway enrichment analysis of nitrogen metabolism in H9N2 AIV infection group, as compared with the mock group. **(B)** Schematic diagram of the process of knockout bacterial genes using Red/ET recombinant engineering technology and a site-specific system. **(C)** Identification of *E. coli moA* mutant (Δ*moaA*) by RT-qPCR. M represents the 5000 DNA marker, + the positive control of wild type *E. coli* (EcN WT; size, 840 bp), and 1-23 the recombinants of EcN moaA (lox71-cm-lox66; size, 1057 bp). **(D)** Identification of recombinant replacement of *EcN napA* gene. M represents the 3000 DNA marker, 1-20 the recombinants of EcN *napA* (lox71-cm-lox66; size, 1071 bp), and 21-22 the positive control of EcN WT (size, 2354 bp). **(E)** Identification of resistance gene deletion of EcN *napA* mutant (Δ*napA*) by RT-qPCR. M represents the 3000 DNA marker, 1-20 the knockout of resistance gene deletion (Δ*napA*; 243 bp), 21-22 the recombinants of EcN *napA* (lox71-cm-lox66; 1071 bp) and 23 the positive control of EcN WT (2,354 bp). **(F)** Recombinant replacement identification of EcN Δ*napA narZ.* M represents the 3000 DNA marker, 1-23 the recombinants of EcN Δ*napA narZ* (lox71-cm-lox66; 1071 bp), 24 the positive control of EcN WT (3,608 bp). **(G)** Identification of resistance gene deletion of EcN Δ*napA* Δ*narZ* by RT-qPCR. M represents the 3000 DNA marker, 1-22 the knockout of resistance gene deletion (Δ*napA* Δ*narZ*; 244 bp), 23 the recombinants of EcN Δ*napA* Δ*narZ* (lox71-cm-lox66; 1071 bp), 24 the positive control of EcN WT (3608 bp). **(H)** Recombinant replacement identification of EcN Δ*napA* Δ*narZ*Δ*narG.* M represents the 3000 DNA marker, 1-21 and 22-42 the recombinants of EcN Δ*napA* Δ*narZ*Δ*naG* (lox71-cm-lox66), 10, 16 and 39 the successfully constructed EcN Δ*napA* Δ*narZ narG* (1082 bp), + the positive control of EcN WT (3622 bp). **(I)** Identification of resistance gene deletion of EcN Δ*napA* Δ*narZ* Δ*narG* by RT-qPCR. The size of the positive control of EcN WT for EcN Δ*napA*, EcN Δ*napA narZ* and EcN Δ*napA* Δ*narZ*Δ*narG* was 2,354, 3,608 and 3,622 bp. The size of EcN Δ*napA*, EcN Δ*napA narZ and* EcN Δ*napA* Δ*narZ*Δ*narG* was 243, 244 and 1,082 bp. M represents the 5000 DNA marker and - the negative control. **(J)** Mutant Competitiveness Index. Data are presented as the mean ± standard deviation (*n* = 3). The differences between groups were analyzed using ANOVA. **P < 0.01. EcN, *E. coli* Nissle1917; *E. coli, Escherichia coli.*

Nitrogen is an essential element for living organisms, and nitrate is one of the most important forms of nitrogen (Cabello et al., 2004). Metabolic pathway enrichment analysis showed that the nitrogen metabolism pathway was positively correlated with H9N2 AIV infection (Figure 7A), further analysis showed that L-Glutamic acid was significantly upregulated while L-Glutamine was significantly downregulated in the nitrogen metabolism pathway. Since that we have revealed that the L-Glutamic acid/L-Glutamine ratio was positively correlated with *Escherichia* (Figure 5D), we speculated that the nitrogen metabolism pathway may play an important role in the proliferation of *Escherichia.* Since a previous study showed that nitrate promotes the growth of *E. coli* in the inflamed gut (Winter et al., 2013), and it was confirmed in the present study that the H9N2 AIV infection could induce inflammation (Figure 6), moreover, nitrate is a key regulator in the nitrogen metabolism pathway which was positively correlated with H9N2 AIV infection (Figure 7A), it was speculated that the proliferation of *E. coli* caused by H9N2 AIV infection may be associated with nitrate.

It has been reported that *MOA* and *AZG* are the key genes for the proliferation of *E. coli* using nitrate (Winter et al., 2013). Next, EcN Δ*moaA* and Δ*AZG* mutants were further constructed according to the basic process for constructing a mutant strain (Figures 7B–I). The CI of EcN:Amp with EcN Δ*moaA* and EcN Δ*AZG* strains was further determined. The results showed that, under anaerobic conditions, the growth competitiveness index of the EcN:Amp and function-deficient strains tended to be 1, indicating that the growth rates of the 2 strains were similar in the same culture system (Figure 7J). After the addition of NO_3_^–^ to the culture system, the CI between the EcN:Amp and EcN Δ*moaA* strains, or between the EcN:Amp and EcN Δ*AZG* strains significantly increased (Figure 7J); this indicated that the growth rate of EcN:Amp was significantly faster than that of the two gene-deficient bacteria, illustrating that EcN:Amp could markedly proliferate with NO_3_^–^.

### Nitrate Promotes the Proliferation of *E. coli in vivo*

**FIGURE 8:**
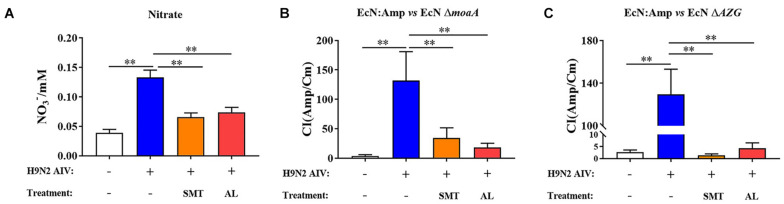
Verification of nitrate metabolism *in vivo*. **(A)** Nitrate content of intestinal epithelium. SMT is an iNOS inhibitor added to evaluate the nitrate content produced by intestinal epithelium. **(B)** CI of EcN:Amp and EcN *moaA*. **(C)** CI between EcN:Amp and EcN *AZG.* Data are presented as the mean ± standard deviation (*n* = 3). The differences between groups were analyzed using ANOVA. **P < 0.01. SMT, S-methyl-isothiourea; iNOS, nitric oxide synthase; CI, competitive index; EcN, *E. coli* Nissle1917.

In order to explore whether the H9N2 AIV infection can induce the production of nitrate in intestinal epithelial cells and promote the proliferation of *E. coli*, an iNOS inhibitor, SMT, was used to evaluate the content of nitrate in the intestinal epithelium. The results showed that the nitrate content in the intestinal epithelium increased significantly following H9N2 AIV infection (*P* < 0.01), while SMT could significantly reduce the H9N2 AIV-induced nitrate content (*P* < 0.01). AL could also significantly reduce the production of H9N2 AIV-induced nitrate (*P* < 0.01, Figure 8A), indicating that H9N2 AIV infection could induce the production of nitrate in intestinal epithelial cells and that AL exerted the effects of an iNOS inhibitor.

The *in vitro* functional verified strains EcN:Amp and EcN Δ*moaA*, and EcN:Amp and EcN Δ*AZG* were fed to SPF chickens at 1:1, and the CI of the 2 pairs of strains was determined to evaluate the intestinal epithelial environment. As shown in Figure 8B, following H9N2 AIV infection, the CI of EcN:Amp and EcN Δ*moaA* increased significantly, indicating that the intestinal environment can produce growth factors that can lead to EcN:Amp growth, but cannot meet the EcN Δ*moaA* growth requirements. Both SMT and AL reduced the CI, indicating that this growth factor was nitrate. Further experiments showed that, following H9N2 AIV infection, the CI of EcN:Amp and EcN Δ*AZG* significantly increased, since the EcN Δ*AZG* strain was a nitrate deficient strain, which, again, indicated that H9N2 AIV infection could induce the production of nitrate in the intestinal epithelium and provide an electron acceptor for the growth of *E. coli*. Following H9N2 AIV infection, both SMT and AL could significantly reduce the CI between strains (Figure 8C), indicating that AL exerted an effect associated with iNOS inhibitors, such as SMT.

In combination, the results showed that H9N2 AIV infection can promote the production of nitrate in intestinal epithelial cells, nitrate can provide electron acceptors for the growth of *E. coli* in the intestine, and AL can inhibit the production of nitrate in the intestinal tract.

## Discussion

The host and intestinal microorganisms are symbiotes, and pathogen infection affects changes in intestinal microorganisms. In recent years, the association between influenza viruses and intestinal microorganisms has attracted considerable attention. For example, studies have shown that the number of *Proteobacteria* increases in the feces of mice infected with the influenza virus (mainly *Enterobacteriaceae*) (Deriu et al., 2016). Subsequent studies have shown that influenza virus infection can change the composition of intestinal microorganisms in mice and cause intestinal microbial imbalance (Yildiz et al., 2018). In 2015, it was reported that H7N9 viral infection caused changes in the composition of human intestinal microorganisms, in antibiotic and non-antibiotic treated patients, the abundance of *E. coli* ranked first and third, respectively (Qin et al., 2015; Groves et al., 2018). However, there are few reports on intestinal microbial disorders caused by H9N2 infection in poultry. It was found that H9N2 AIV can disrupt the composition of intestinal microorganisms, and viral infection can induce intestinal microbiota to regulate the immune response mediated by type I interferon, and then play an antiviral role (Yitbarek et al., 2018a, b,c). In the present study, it was found that intestinal *E. coli* rapidly proliferated and the intestinal mucosal barrier was damaged following H9N2 AIV infection in SPF chickens, which was consistent with the previous research results of our group (Li et al., 2018).

The destruction of the intestinal barrier increases intestinal permeability, and intestinal bacteria are easy to translocate through the barrier to visceral organs (Ma et al., 2018). In addition, certain studies have shown that HIV infection causes systemic microbial disorder and increased microbial translocation in the plasma (Xu et al., 2018). Therefore, it was speculated that the increased *E. coli* in the intestinal tract caused by H9N2 AIV may translate to other organs. As expected, our results showed that bacteria could indeed be isolated from the liver and other visceral tissues following H9N2 AIV infection. In order to further confirm whether the microbiota in the viscera were translocated from the intestinal tract, NeonGreen-tagged bacteria were administrated into the gastrointestinal tract and used to trace the translocation of *E. coli* from the intestinal tract to the mesentery, lung and liver, in order to illustrate whether the bacterial secondary infection in SPF chickens infected with H9N2 was due to intestinal flora disorder and then result to the *E. coli* translocate to the visceral organs. Furthermore, the NeonGreen-tagged *E. coli* bacteria count in different treatment groups was detected by RT-qPCR to further illustrate that H9N2 AIV infection causes NeonGreen-tagged *E. coli* translocation, leading to bacterial secondary infection. This explains the causal relationship among H9N2 virus, intestinal microbial imbalance and bacterial secondary infection.

H9N2 AIV infection damages the intestinal mucosal barrier in chickens, resulting in the increase of *E. coli* and its translocation to other organs. Therefore, in-depth understanding of the causes of *E. coli* proliferation is key to exploring the mechanism of bacterial secondary infection. *E. coli* is a facultative anaerobic bacteria that belongs to the *Proteobacteria* of the *Escherichia* genus. *E. coli* is a conditional pathogen in the intestinal tract and has a symbiotic relationship with the body. Under normal circumstances, *E. coli* is harmless to the body; however, when a disease occurs in the host, *E. coli* proliferates to become harmful bacteria. *Proteobacteria*, as a feature of intestinal flora imbalance, is often associated with some inflammatory diseases, such as ulcerative colitis (Carvalho et al., 2012; Maharshak et al., 2013). This phenomenon suggests that the proliferation of facultative anaerobes in the intestinal tract may be driven by oxygen or other respiratory electron receptors, thus putting forward the “oxygen hypothesis” (Rigottier-Gois, 2013). In the colitis animal model group, the decrease of intestinal short-chain fatty acids caused intestinal epithelial cells to grow on glycolysis and lactic acid fermentation (Kelly et al., 2015; Byndloss et al., 2017). As a result, intestinal epithelial cells released lactate into the intestinal cavity and the epithelial cells did not consume oxygen, causing oxygen molecules to be released into the intestinal cavity and the intestinal tract to become a micro-oxygen environment and promote the proliferation of *E. coli* (Takaishi et al., 2008; Hughes et al., 2017). Symbiotic *Enterobacteriaceae* can use formate as an electron donor and oxygen as an electron acceptor for respiratory metabolism (Hughes et al., 2017). In addition, *Enterobacteriaceae* can use the nitrate produced by the host as an electron acceptor. Under healthy conditions, *E. coli* is a symbiotic bacteria in the intestine. When inflammation occurs, the abundance of *E. coli* in intestinal tract significantly increased and use nitrate for metabolic competitive growth, which is an independent metabolic pathway (Winter et al., 2013). Therefore, there is an important association among the imbalance of intestinal flora, the proliferation of *Enterobacteriaceae* and the metabolite.

The present study was the first to perform intestinal metabonomics to study the changes of metabolites in the intestinal lumen following H9N2 AIV infection. It was found that the L-Glutamic acid/L-Glutamine ratio was significantly positive correlated with H9N2 infection group, as compared with the mock group. Further correlation analysis showed that *E. coli* was correlated with the L-Glutamic acid/L-Glutamine ratio. Combined with metabonomic pathway enrichment analysis, it was found that the metabolic pathways associated with L-Glutamic acid and L-Glutamine was nitrogen metabolism. It has been reported that *IFN-*γ can induce the expression of the *NOS2* gene in the intestinal epithelium and can encode inducible *iNOS*, while *iNOS* can catalyze the formation of nitric oxide from arginine in the intestinal tract (Salzman et al., 1996). According to the metabonomics results, it was inferred that the imbalance of intestinal bacteria caused by the H9N2 AIV infection may have been caused by the anaerobic metabolism and proliferation of *Enterobacteriaceae* using nitrate as an electron acceptor.

In order to test that hypothesis, interferon and other genes, as well as nitrate content, were first determined in the intestinal epithelium. Our results showed that H9N2 AIV could upregulate the mRNA level of *TNF-*α, *IFN-*γ and *iNOS*, and the nitrate content in the intestinal epithelium was also significantly increased, indicating that *E. coli* may proliferate through nitrate metabolism in the inflamed intestinal tract. In order to determine the association of nitrate metabolism and proliferation of *E. coli* in chickens, nitrate-deficient mutant *E. coli* was constructed by genetic engineering recombination technology, and the metabolic environment in the intestinal epithelium was judged by the growth of functional deficient strains. The present study showed that H9N2 AIV infection can increase the growth competition index between normal nitrate metabolic and mutant strains, indicating that *E. coli* in the intestine proliferates markedly using nitrate produced by the intestinal epithelium as a respiratory electron. Therefore, this study elucidated the mechanism of secondary *E. coli* infection in chickens caused by H9N2 AIV infection that H9N2 AIV infection induces inflammation in the intestinal mucosa and further promotes the secretion and release of nitrate from the host intestinal epithelium, *E. coli* in the intestine proliferates massively with nitrate as the electron acceptor and the *E. coli* in the intestine translocate to visceral tissue through the damaged intestinal barrier, resulting in secondary bacterial infection. The intestinal microorganism plays an important role in innate immune response in poultry, and therefore the regulatory relationship among *E. coli*, nitrate and innate immune needs to be further studied.

In the breeding industry, the highly prevalent abuse of chemical drugs and antibiotics poses a severe threat to humans. The emergence of super resistant strains, livestock and poultry products, drug residues, and environmental pollution is becoming an increasingly serious problem. Traditional Chinese herbal medicines have been widely used as sources of novel antiviral drugs. For that reason, the present study used the Chinese medicine AL to regulate secondary bacterial infection caused by H9N2 AIV. Our results showed that AL could regulate the changes in intestinal bacteria, restore the dominant position of *Lactobacillus*, and inhibit the bacterial disorder caused by H9N2 AIV, and particularly the abnormal proliferation of *E. coli*. It has been reported that Agastache rugosa inhibits the expression of *iNOS* in osteocytes activated by *TNF-*α and *IL-1*β (Oh et al., 2005). Our results of this study indicated that AL can alleviate intestinal damage, reduce inflammation and *iNOS* expression, and then reduce the production of nitrate in intestinal epithelial cells. In general, AL can regulate the expression of inflammatory genes in the intestinal epithelium of the host, and then regulate the composition of intestinal microorganisms and inhibit the abnormal proliferation of *E. coli* by inhibiting metabolite nitrate. Furthermore, AL can repair intestinal mucosal damage and provide a strong protection for *E. coli* translocation.

## Conclusion

In conclusion, the present study firstly revealed the mechanism of H9N2 AIV-induced secondary *E. coli* infection. In briefly, H9N2 AIV infection caused intestinal microbial disorder and made *Escherichia* become the dominant flora, promoted bacterial translocation from intestinal tract to visceral tissue through the damaged intestinal barrier, induced inflammation in the intestinal mucosa and promoted the secretion and release of nitrate from the host intestinal epithelium. Importantly, the nitrate was the reason for the growth of *E. coli* in the inflammatory intestinal tract following H9N2 AIV infection. In addition, AL can restore the homeostasis of the intestinal flora, improve the injury degree of intestinal epithelial mucosa, inhibit inflammatory genes and the production of nitrate in the intestinal epithelium, effectively preventing the proliferation and translocation of *E. coli* in the intestine. This study provided effective theoretical guidance for the prevention and control of H9N2 AIV-induced secondary bacterial infection.

## Data Availability Statement

The original contributions presented in the study are publicly available. This data can be found here: https://www.ncbi.nlm.nih.gov/bioproject/?term=PRJNA625215.

## Ethics Statement

The animal study was reviewed and approved by the South China Agricultural University Committee of Animal Experiments (approval ID, SYXK-2014-0136). Written informed consent was obtained from the owners for the participation of their animals in this study.

## Author Contributions

XZ, HL, and WL conceived study, designed the experiments, and analyzed the data. QZ, CW, ZX, and XC performed the experiments and analyzed the data. XZ wrote the manuscript. HZ and QX supervised the experiments and edited the manuscript. All authors contributed to the article and approved the submitted version.

## Conflict of Interest

The authors declare that the research was conducted in the absence of any commercial or financial relationships that could be construed as a potential conflict of interest. The reviewer WR declared a shared affiliation, with no collaboration, with the authors to the handling editor at the time of review.
