# Supplementary material for: Nitrate Is Crucial for the Proliferation of Gut Escherichia coli Caused by H9N2 AIV Infection and Effective Regulation by Chinese Herbal Medicine Ageratum-Liquid
Source: Front Microbiol. 2020 Oct 30;11:555739. doi: 10.3389/fmicb.2020.555739 (PMC7662154; doi:10.3389/fmicb.2020.555739)
Supplement: Supplementary file 1 [file Data_Sheet_1.docx]

**Nitrate is crucial for the proliferation of gut** **[Escherichia](javascript:;)**[**coli**](javascript:;)**caused by H9N2 AIV infection and effective regulation by Chinese herbal medicine ageratum-liquid**

Xinheng Zhang ^1,2,3,4^, Qiqi Zhao ^1,3,4^, Che Wu ^1,4^, Zi Xie ^1,3,4^, Xiaotong Ci ^1,4^, Hongxin Li ^1,3,4^, Wencheng Lin ^1,2,3,4^, Huanmin Zhang ^5^, Qingmei Xie ^1,2,3,4*^

**Supplementary Figures and Tables**

**Supplementary Figures**


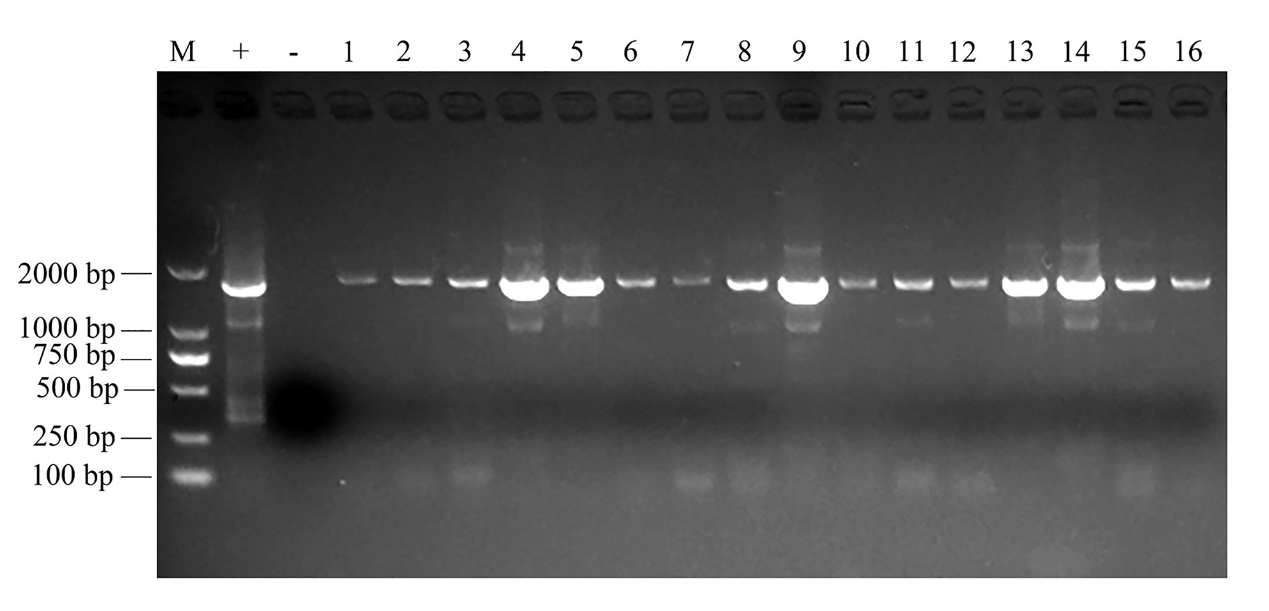


**Supplementary Figure S1.** Detection of HA gene of H9N2 AIV in cloacal by RT-qPCR. Cloacal swabs were collected on day 3 after infection by RT-qPCR to detect the HA gene of H9N2 AIV in H9N2 AIV infection group and H9N2 AIV infection with feeding AL group. M represents the 2000 DNA marker, + the positive control of H9N2 AIV, - the negative control, and 1-16 the cloacal swab sample, 1-8 represents the cloacal swab sample in H9N2 AIV infection group, 9-16 represents the cloacal swab sample in H9N2 AIV infection with feeding AL group. Eight repetitions for samples in each group. HA, hemagglutinin.


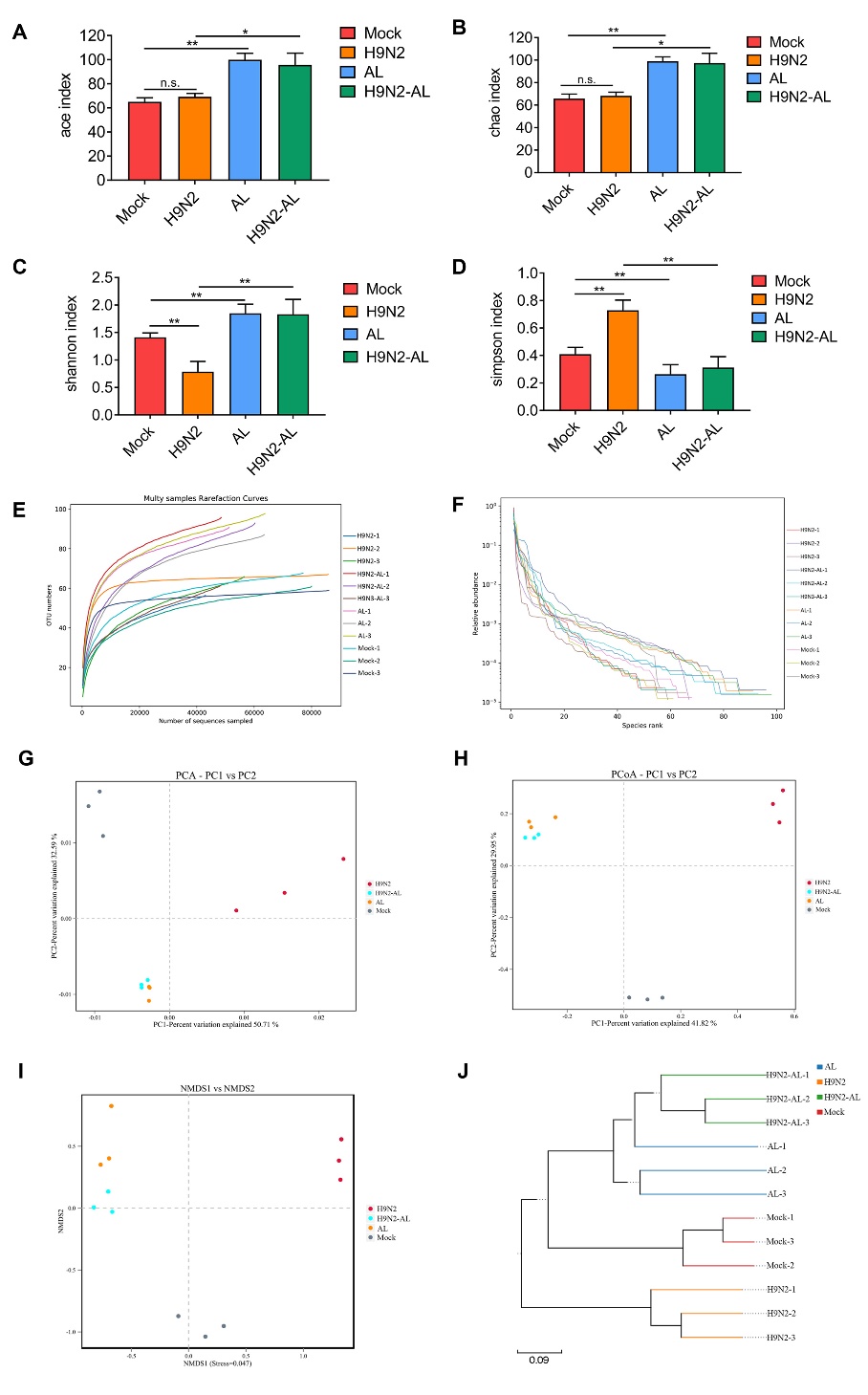


**Supplementary Figure S2.** α Diversity index statistics and β diversity analysis of gut microbiota 5 days after H9N2 AIV infection and AL treatment in SPF chickens. Statistical results of **(A)** Ace, **(B)** Chao, **(C)** Shannon and **(D)** Simpson of α diversity indexes in different treatment groups. **(E)** Dilution and **(F)** grade abundance curves based on OTU analysis in different treatment groups. **(G)** PCA analysis map, **(H)** PCoA analysis map of Bray-Curtis algorithm, **(I)** NMDS analysis map of the Bray-Curtis algorithm and **(J)** sample UPGMA clustering tree the Bray-Curtis algorithm of β diversity analysis based on OTU in different treatment groups. Data are presented as the mean ± standard deviation (n=3). The differences between groups were analyzed using ANOVA. *P<0.05 and **P<0.01. n.s., not significant. AL, ageratum-liquid; PCA, principal component analysis; PCoA, principal coordinates analysis; NMDS, non-metric multidimensional scaling; UPGMA, unweighted pair group method with arithmetic mean; OTU, operational taxonomic unit (OTU).


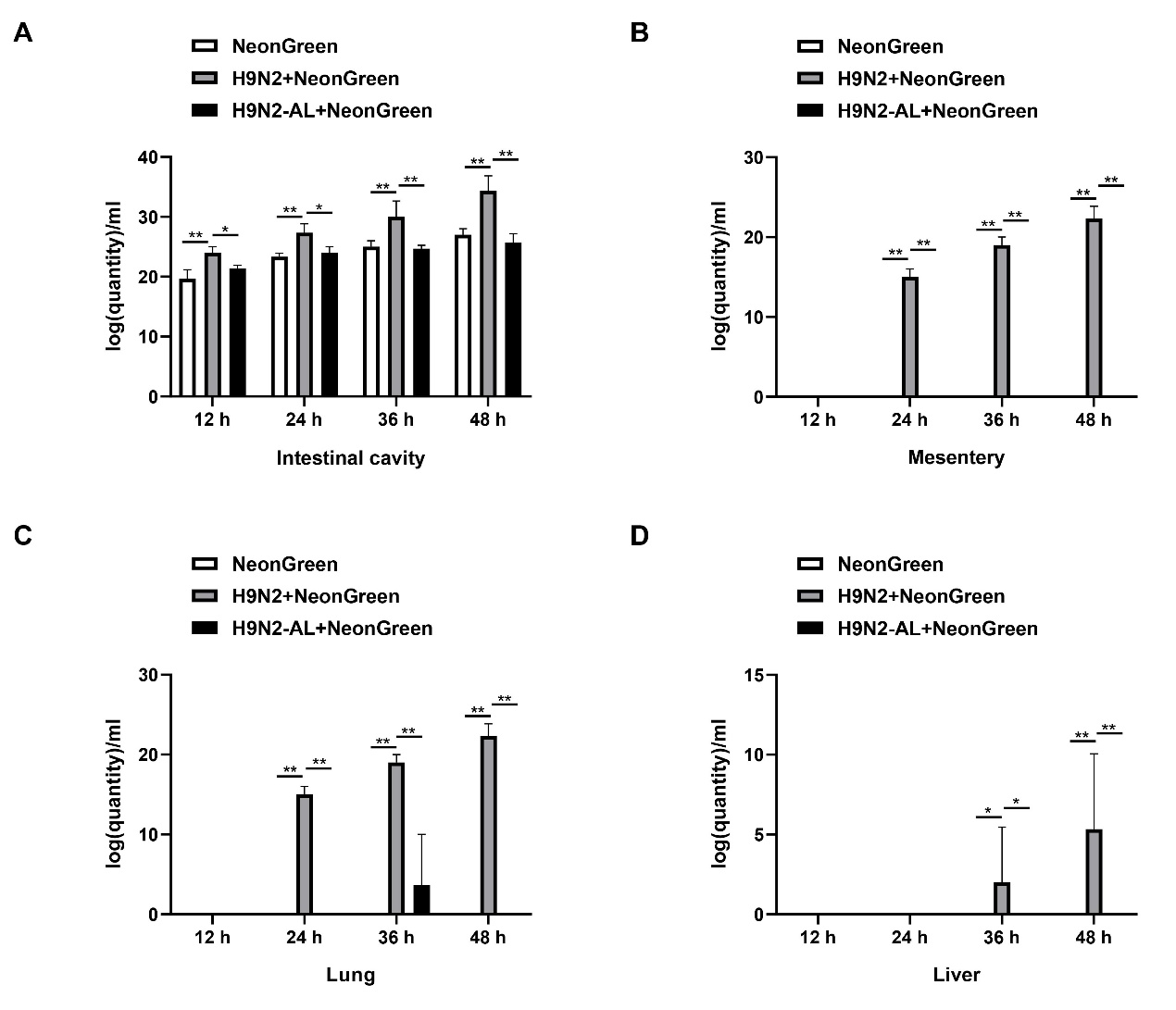


**Supplementary Figure S3. Quantity of *E. coli* in enteric contents, mesentery, lung and liver by RT-qPCR.** (A) Quantity of *E. coli* in enteric contents from chickens in NeonGreen, H9N2-NeonGreen, and H9N2-AL-NeonGreen groups at 12 h, 24 h, 36 h and 48 h. (B) Quantity of *E. coli* in mesentery from chickens in NeonGreen, H9N2-NeonGreen, and H9N2-AL-NeonGreen groups at 12 h, 24 h, 36 h and 48 h. (C) Quantity of *E. coli* in lung from chickens in NeonGreen, H9N2-NeonGreen, and H9N2-AL-NeonGreen groups at 12 h, 24 h, 36 h and 48 h. (D) Quantity of *E. coli* in liver from chickens in NeonGreen, H9N2-NeonGreen, and H9N2-AL-NeonGreen groups at 12 h, 24 h, 36 h and 48 h. Data are presented as the mean ± standard deviation (n=3). The differences between groups were analyzed using ANOVA. *P<0.05 and **P<0.01. AL, ageratum-liquid.


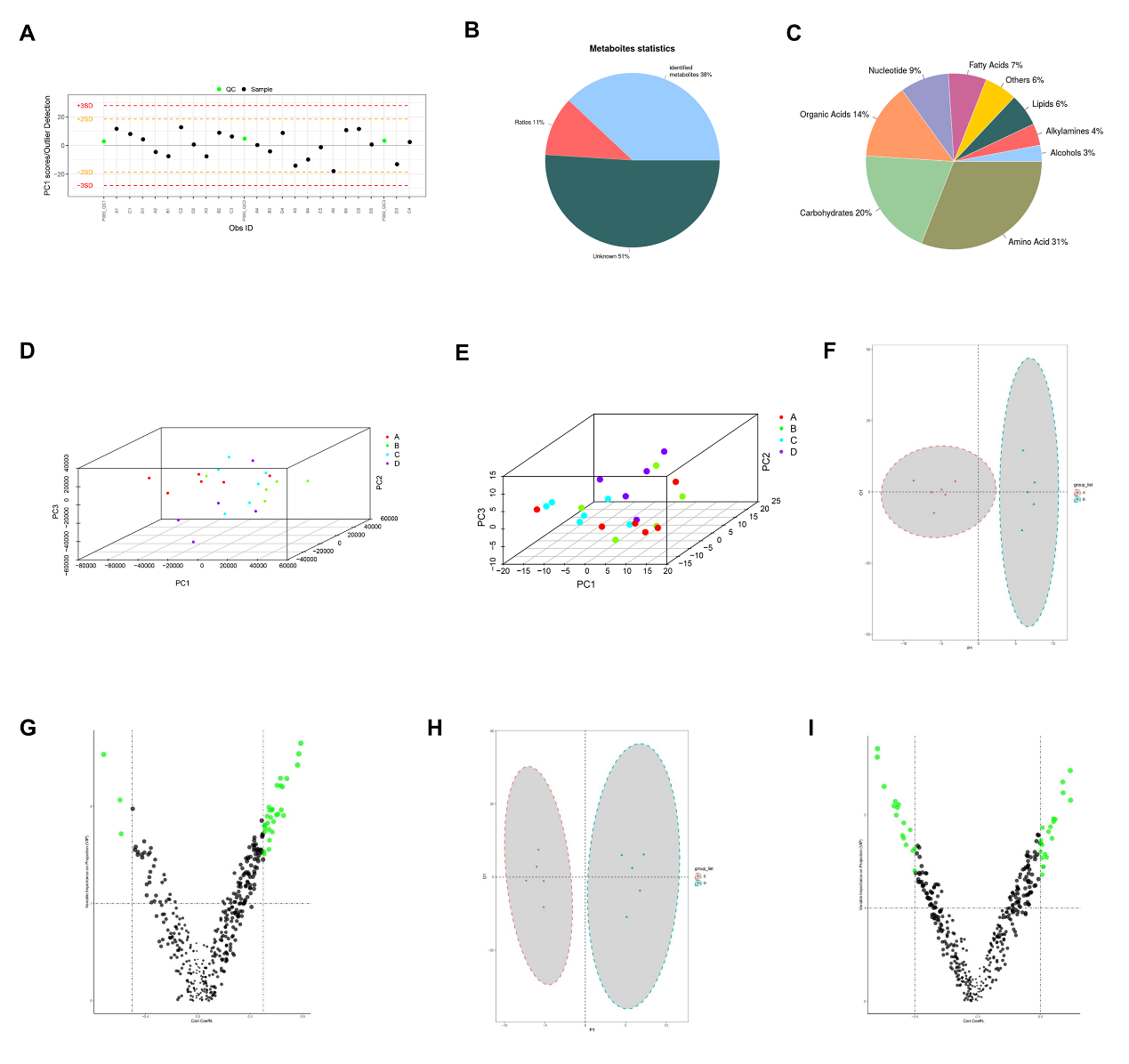


**Supplementary Figure S4.** Effect of H9N2 AIV infection and AL treatment on intestinal metabolism of SPF chickens 5 days post-infection. **(A)** Data multivariate control chart of ileum content samples from different treatment groups. Each dot represents the summary of global metabolite profiles of each individual. Green dots denote the pooled QC samples and black dots the actual samples. Scores that exceed ±3S.D. were generally considered as the risk of outliers. **(B)** Metabolite statistics detected in the samples of different treatment groups. **(C)** Metabolite classes and compositions detected in the samples of different treatment groups. **(D)** Overview of metabolic profiles of all samples, as revealed by 3D-PCA Score Plot. **(E)** 3D-PLS-DA Score Plot revealing classifications of the samples in different treatment groups. **(F)** Visualization of overall metabolite profile difference between mock (A group) and H9N2 infection (B group) groups, as shown by the OPLS-DA predictive/discriminate score plot. **(G)** Visualization of differential metabolite profiles using Vplot between the mock (A group) and H9N2 infection (B group) groups. **(H)** Visualization of overall metabolite profile difference between the H9N2 infection (B group) and after H9N2 infection with feeding AL (D group) groups, as showed by the OPLS-DA predictive/discriminate score plot. **(I)** Visualization of differential metabolite profiles using vplot between the H9N2 infection (B group) and after H9N2 infection group with feeding AL (D group) groups. A, mock group; B, H9N2 AIV infection group; C, AL feeding group; D, after H9N2 infection group with feeding AL group. The number of sequencing samples in each group was ≥5. AL, Ageratum-liquid; SPF, specific pathogen free; PCA, principal component analysis; OPLS-DA, Orthogonal Projections to Latent Structures Discriminant Analysis.

**Supplementary Tables**

**Supplementary Table S1.** Isolation of NeonGreen-tagged bacteria in various tissues and organs of different treatment groups.

|  | NeonGreen | | | | H9N2 + NeonGreen | | | | H9N2-AL + NeonGreen | | | | |
| --- | --- | --- | --- | --- | --- | --- | --- | --- | --- | --- | --- | --- | --- |
|  | 12 h | 24 h | 36 h | 48 h | 12 h | 24 h | 36 h | 48 h | 12 h | 24 h | 36 h | 48 h |  |
| Intestinal cavity | **+**  **(3/3)** | **+**  **(3/3)** | **+**  **(3/3)** | **+**  **(3/3)** | **+**  **(3/3)** | **+**  **(3/3)** | **+**  **(3/3)** | **+**  **(3/3)** | **+**  **(3/3)** | **+**  **(3/3)** | **+**  **(3/3)** | **+**  **(3/3)** |  |
| Mesentery | -  (3/3) | -  (3/3) | -  (3/3) | -  (3/3) | -  (3/3) | **+ (3/3)** | **+**  **(3/3)** | **+ (3/3)** | -  (3/3) | -  (3/3) | -  (3/3) | -  (3/3) |  |
| Lung | -  (3/3) | -  (3/3) | - (3/3) | - (3/3) | -  (3/3) | **+ (3/3)** | **+**  **(3/3)** | **+ (3/3)** | -  (3/3) | -  (3/3) | **+**  **(1/3)** | -  (3/3) |  |
| Liver | -  (3/3) | -  (3/3) | -  (3/3) | -  (3/3) | -  (3/3) | - (3/3) | **+**  **(1/3)** | **+ (2/3)** | -  (3/3) | -  (3/3) | -  (3/3) | -  (3/3) |  |

**Supplementary Table S2.** The differential metabolites were screened out in the H9N2 AIV infection with feeding AL group, as compared with the H9N2 AIV infection group (log2FC > 1.5, *P* Value < 0.05, VIP > 1).

|  | | | |
| --- | --- | --- | --- |
| Name | VIP | Corr | *P* Value |
| L-Alanine | 2.1441 | -0.6556 | 0.0429 |
| Urea | 2.6190 | -0.7984 | 0.0067 |
| Guanine | 2.4746 | 0.7359 | 0.0172 |
| Ratio of Uric acid/Xanthine | 2.7098 | -0.7971 | 0.0068 |
| Niacinamide | 2.1566 | 0.7381 | 0.0167 |
